# Clinical study of Jianpi Qingchang decoction in the treatment of ulcerative colitis patients with spleen deficiency and dampness-heat syndrome accompanied by fatigue: Study protocol for a randomized controlled trial

**DOI:** 10.1016/j.conctc.2024.101409

**Published:** 2024-12-05

**Authors:** Zi-Xuan Liu, Xiao-Yan Liu, Wei-wei Tan, Wei-Bing Zhang, Ya-Li Zhang, Lie Zheng, Yan-Cheng Dai

**Affiliations:** a Department of Gastroenterology, Shanghai Traditional Chinese Medicine-Integrated Hospital, Shanghai University of Traditional Chinese Medicine, Shanghai, 200082, China; b Institute of Digestive Diseases, Long Hua Hospital, Shanghai University of Traditional Chinese Medicine, Shanghai, 200032, China; c Department of Gastroenterology, Traditional Chinese Medicine Hospital of Shanxi Province, Xi'an, 710003, China

**Keywords:** Ulcerative colitis, Spleen deficiency and dampness-heat syndrome, Jianpi Qingchang decoction, Fatigue, Randomized controlled trial

## Abstract

**Background:**

Ulcerative colitis (UC) is a chronic non-specific inflammatory intestinal disease, categoried under "dysentery" and "intestinal bleeding" in Traditional Chinese Medicine (TCM). Jianpi Qingchang decoction (JPQC) is a combination formula specifically designed for the treatment of UC. The primary objective of this study is to examine the clinical efficacy of JPQC in individuals diagnosed with UC who exhibit both spleen deficiency and dampness-heat syndrome, along with the presence of fatigue. The investigation will focus on assessing the impact of JPQC on the gut microbiota and metabolites in these patients, aiming to elucidate the regulatory mechanism that JPQC exerts on the gut microbiota and metabolites in the context of UC-related fatigue.

**Methods:**

In this randomized clinical trial, 140 subjects diagnosed with UC will be recruited and randomized into two groups. They will receive either JPQC combined with mesalazine or mesalazine alone for 12 weeks. Follow-up visits will be conducted every four weeks, with a post-treatment visit scheduled at 6 months. The primary outcome measures include the Inflammatory bowel disease fatigue scale(IBD-F). Secondary efficacy indicators comprise the assessment of TCM syndrome and individual syndrome efficacy before and after treatment, Modified Mayo score, Simple clinical colitis activity index (SCCAI), as well as the Inflammatory Bowel Disease Questionnaire (IBDQ) for each group. The other outcomes are the Intestinal microbial diversity and non-targeted metabonomics, which will be measured at baseline and 12 weeks after randomization.

**Discussion:**

If effective, JPQC will provide substantial clinical evidence concerning the effectiveness and safety in the treatment of patients with UC experiencing spleen deficiency and dampness-heat syndrome accompanied by fatigue.

**Trial registration:**

ChiCTR2300068348.

## 1 Introduction

Ulcerative colitis (UC) is a chronic, non-specific inflammatory intestinal disease primarily affecting the colorectal mucosa and submucosa, categorized under inflammatory bowel disease (IBD). It is estimated that the global prevalence of ulcerative colitis will reach 5 million cases in 2023, with a rising incidence worldwide [1]. The main clinical symptoms of UC include persistent or frequent episodes of diarrhea and mucous purulent and bloody stools. These symptoms may be accompanied by systemic manifestations such as abdominal pain, acute diarrhea, and fatigue.

Fatigue, characterized by a disproportionate lack of energy or exhaustion to physical exertion, adversely affects daily activities and cannot be alleviated by rest [2]. It is a multifaceted, multifactorial symptom associated with various chronic diseases [3]. Notably, Fatigue is a significant clinical symptom for patients with IBD [4], reaching a prevalence of 86 % in those with active IBD and 50 %–52 % in those with mild to inactive IBD [5]. In Chinese patients with UC, the prevalence rate of fatigue is 61.8 %, with higher rates in active disease (68.2 %) than in remission (40.0 %). Factors such as Montreal classification, disease activity, and anxiety are linked to an increased risk of fatigue risk Background [6]. The presence of fatigue in patients with UC is associated with a diminished quality of life [7]. Addressing fatigue is crucial not only for improving the care and quality of life for patients with UC but also for reducing direct medical expenses and the indirect economic impact on work productivity and function [8]. Therefore, it is particularly important to find practical and effective ways to intervene fatigue for improving the care and quality of life of patients with UC.

Traditional Chinese medicine (TCM) posits that the weakness of the spleen and stomach, exogenous pathogens, improper diet, and emotional disorders can contribute to UC. The disease primarily affects the large intestine and is characterized by spleen deficiency, and dampness-heat, blood stasis, and phlegm-dampness. UC is further categorized into seven syndrome types, such as spleen deficiency and dampness accumulation syndrome, large intestine damp-heat syndrome, excessive heat toxin syndrome and liver depression and spleen deficiency syndrome, with spleen deficiency and dampness accumulation syndrome and large intestine damp-heat syndrome being common [9]. In our previous study, we observed varying degrees of fatigue symptoms in patients with damp-heat syndrome of the large intestine and spleen deficiency and dampness accumulation syndrome. Fatigue was notably more prevalent and severe in the latter. Patients with UC and damp-heat syndrome of the large intestine exhibited symptoms such as "stagnation of wetness-evil," characterized by a heavy head, heavy body sleepiness, and sore limbs, among which heavy body sleepiness is its representative symptom. In contrast, patients with UC with spleen deficiency and dampness accumulation syndrome manifested "weakness" symptoms, including listlessness, fatigue, and weakness of limbs, among which fatigue and weakness are its characteristic symptoms. This finding holds significant guidance for clinical syndrome differentiation and the treatment of UC-related fatigue. The methods involving clearing heat and eliminating dampness, invigorating the spleen, and benefiting qi, as explored in this study, offer valuable insights into therapeutic approaches for managing UC-related fatigue [10]. Guided by the principles of invigorating the spleen and qi, clearing away heat and toxic materials, cooling blood and stopping bleeding, and regulating qi and relieving pain, we formulated and optimized Jianpi Qingchang decoction (JPQC) consisting of *Astragalus membranaceus (Huang Qi)30g, Portulaca oleracea (Ma Chi Xian)30g, Codonopsis pilosula (Dang Shen)15g, Coptidis Rhizoma (Huang Lian)3 g, Sanguisorba officinalis (Sheng Di Yu)15g, Panax notoginseng (San Qi)6g, Bletilla striata (Bai Ji)3g, Radix Aucklandiae (Mu Xiang)6g, and Radix Glycyrrhizae (Sheng Gan Cao)6g* [11]. Forty-four phytochemicals, including astragaloside A, oleracein A, and coptisine, were identified from JPQC using the ultra-high performance liquid chromatography-quadrupole time-of-flight mass spectrometry (UPLC-Q-TOF/MS) method [12]. Our previous study revealed that JPQC effectively alleviated clinical symptoms of abdominal pain and diarrhea in patients with mild-moderate UC, improved mental fatigue, and increased quality of life [13,14]. However, there is a limited exploration of the impact of JPQC on fatigue in patients with UC with spleen deficiency and dampness-heat syndrome. Consequently, we have designed a randomized controlled clinical trial to comprehensively investigate the effectiveness and safety of JPQC in treating patients with UC with spleen deficiency and dampness-heat syndrome accompanied by fatigue. This research aims to contribute valuable insights into potential therapeutic interventions for addressing fatigue in patients with UC, particularly those with spleen deficiency and dampness-heat syndrome.

## 2 Materials and methods

### 2.1 Study design

**Fig. 1:**
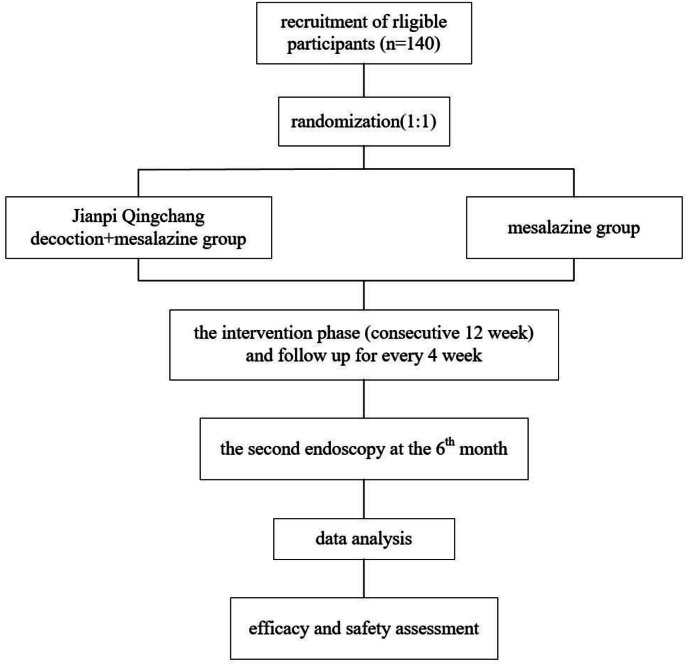
The flow chart of the trial.

The research protocol obtained approval by the Ethics Committee of Shanghai Hospital of Integrated Traditional Chinese and Western Medicine affiliated with Shanghai University of Traditional Chinese Medicine (2022-053-1) and was registered in the Chinese Clinical Trial Registry on February 15th, 2023 (ChiCTR2300068348). The flow chart of the trial is depicted in Fig. 1.

This is a randomized controlled multicenter clinical trial comprising two parallel groups. From January 2024 to June 2025, 140 patients meeting the diagnostic criteria and syndrome types of UC will be recruited. During the recruitment process, potential participants meeting the inclusion criteria will be thoroughly informed about the study's purpose, examinations, intervention measures, and follow-up procedures. Upon obtaining informed consent, eligible subjects will be randomly assigned to two groups in a 1:1 ratio. The groups will receive either JPQC combined with mesalazine or mesalazine intervention for 12 weeks. The UC patients will be followed up again after 6 months. The schedule details are listed in Table 1.

**Table 1:** Schedule of trial.

Period	enrollment	baseline	treatment			follow-up
time points	week-1	week 0	week 4	week 8	week 12	week 24
patients						
Informed consent	✓					
Inclusion/Exclusion criteria	✓					
Medical history	✓					
Physical examination	✓					✓
Laboratory examination	✓					✓
Randomization	✓					
Intervention						
Jianpi Qingchang decoction(JPQC) group	JPQC:two times daily; + mesalazine: 1g/three times daily					
Mesalazine control group	1g/three times daily					
Outcomes						
Inflammatory bowel disease fatigue scale(IBD-F)		✓	✓	✓	✓	✓
Inflammatory Bowel Disease Questionnaire (IBDQ)		✓	✓	✓	✓	✓
TCM symptom efficacy		✓	✓	✓	✓	✓
Modified Mayo score		✓			✓	✓
Simple clinical colitis activity index (SCCAI)		✓				
Intestinal microbial diversity and non-targeted metabonomics		✓			✓	
Trial evaluation						
Safety of medicine		✓	✓	✓	✓	✓
Adverse event		✓	✓	✓	✓	✓
Reasons of drop-outs or withdrawals		✓	✓	✓	✓	
Patient's compliance					✓	

### 2.2 Participants

#### 2.2.1 Recruitment

Patients will be recruited from the outpatient and inpatient gastrointestinal endoscopy departments of Shanghai Hospital of Integrated Traditional Chinese Medicine, Longhua Hospital affiliated with Shanghai University of TCM and Traditional Chinese Medicine Hospital of Shaanxi Province.

#### 2.2.2 Inclusion criteria

1) Age between 18 and 70 years old, both male and female.
2) Diagnosis of ulcerative colitis confirmed by electronic colonoscopy and pathology, meeting the criteria established by the consensus on the diagnosis and treatment of inflammatory bowel disease (2018 Beijing) [15].
3) Adherence to the 2018 Guidelines for Diagnosis and Treatment of Ulcerative Colitis, a Common Disease of Digestive System [16], TCM Syndrome Differentiation Criteria for spleen deficiency and dampness-heat syndrome.
4) Underwent enteroscopy within the past 1 year.
5) Absence of serious heart, liver, kidney, and other significant organ and blood system diseases.
6) Full understanding of the purpose and content of the test, good compliance, and voluntary signing of the informed consent form.

#### 2.2.3 Exclusion criteria

1) Participation in other clinical trials in the past 6 months.
2) Use of antibiotics in the last 3 months.
3) Use of immunosuppressants, biological agents, other non-steroidal (steroidal) anti-inflammatory drugs, gastrointestinal mucosal protective agents, intestinal probiotics and probiotics in the past 4 weeks.
4) Pregnant or lactating women.
5) Presence of gastric and duodenal ulcers, history of intestinal surgery, intestinal obstruction, intestinal perforation, perianal abscess, severe hemorrhagic disease, or tumor.
6) Patients with mental illness, are unable to control themselves, or lack autonomy.
7) Lack of legal capacity or limited legal capacity.
8) History of other diseases estimated by researchers to interfere with trial results or increase patient risk.
9) Any other circumstances deemed unsuitable for participating in the study by the researcher.

#### 2.2.4 Termination criteria

1) Incomplete or imperfect data.
2) Unwillingness to cooperate with or failure to complete the clinical study.

#### 2.2.5 Withdrawal criteria

1) Poor compliance with medication, not adhering to the required medication schedule.
2) Loss of follow-up, patients who do not cooperate with reexamination and return visit
3) Self-withdrawal from clinical observation for personal reasons.
4) Occurrence of allergies or serious adverse reactions during taking medicine.

#### 2.2.6 Sample sizes

Based on the literature findigns, the control group treated with mesalazine showed a 12-week effective rate of 71 % [17], while the experimental group treated with JPQC recipe and mesalazine exhibited an effective rate of 94.12 % in 12 weeks [18]. Considering a clinical difference to improve the effective rate, with α set at a unilateral level of 0.05 and β at 0.10, the sample size calculation was performed using the formula

$$n=\frac{{2\bar{p}\bar{q}\left(Z_{\alpha }+Z_{\beta }\right)}^{2}}{{\left(p1-p2\right)}^{2}}$$

This yielded a sample size of approximately 56 cases for each group (n1 = n2 ≈ 56 cases). Accounting for a dropout rate of 20 %, the study required a total of 140 cases, with 70 patients in the control group and 70 patients in the test group.

### 2.3 Interventions

#### 2.3.1 The experimental group

The experimental group receiving JPQC combined with mesalazine. JPQC decoction pieces, sourced from the hospital's herbal medicine room, will be administered twice a day, 0.5 h after breakfast and dinner. Mesalazine enteric-coated tablets, 1.0 g per dose, will be given three times a day, 1 h after each meal. The interval between taking JPQC and Mesalazine tablets is 0.5 h.

#### 2.3.2 The control group

The control group receiving mesalazine alone.

### 2.4 Outcomes

#### 2.4.1 Primary outcome

The primary outcome of the study is the inflammatory bowel disease fatigue scale(IBD-F), which will be measured at baseline and 4, 8, 12 weeks after randomization. The IBD-F consists of 3 parts. Possible scores are 0–20 and 0–120 in part I and II, respectively. If the ‘not applicable – N/A’ (not applicable) option is checked (questions 3, 4, 9, 12, 13, 14), a corrected result has to be calculated from the following formula: Corrected result = actual total result/(120 – number of N/As × 4) × 120 [19].

#### 2.4.2 Secondary outcomes

The secondary outcomes include the following items.

##### 2.4.2.1 Inflammatory Bowel Disease Questionnaire (IBDQ)

IBDQ covers four dimensions: intestinal symptoms, systemic symptoms, emotional ability, and social ability, with a total of 32 questions, with 1–7 options for each question, with 1 for the worst and 7 for the best options. The IBDQ is a total score of 4 dimensions, with higher scores representing higher quality of life [20].

##### 2.4.2.2 TCM symptom efficacy

TCM symptom efficacy evaluation criteria will refer to the TCM Symptom Score Table for Gastrointestinal Diseases [21]. The scores of 0, 3, 5, and 7 represented no symptoms, mild, moderate, and severe symptoms, respectively, where the main symptoms included abdominal pain, diarrhea,mucous purulent bloody stool, tenesmus, anal burning, and the secondary symptoms included dry mouth with bitter taste in mouth, poor food intake and poor appetite, fatigue, lazy word.

##### 2.4.2.3 The disease activity

The modified Mayo score [15] and simple clinical colitis activity index (SCCAI) [22] will be recorded to assess the disease activity.

### 2.5 Mechanism outcomes

The procedure involves the detection and analysis of intestinal microbial diversity and non-targeted metabonomics. To conduct this, it is essential to collect freshly excreted feces, preferably from the first bowel movement in the morning. Approximately 2 g of the middle section of the feces should be carefully extracted and deposited into a sterile fecal collection tube. Two distinct fecal samples need to be collected sequentially, with detailed information such as name, sex, age, and sampling time noted for each specimen. These outcomes are measured before and after treatment.

### 2.6 Safety outcomes

The main safety indicators include laboratory tests before and after treatment, encompassing blood routine, urine routine, fecal routine, occult blood, liver and kidney function, and electrocardiogram.

### 2.7 Statistical analysis

Statistical analysis will be conducted using SPSS 24.0 software. Descriptive statistics for measurement data will include mean ± standard deviation ($\bar{X}$ ± S) for data conforming to normal distribution. Two independent samples t-tests will be employed for comparing means, and variance analysis for multi-sample comparisons. Nonparametric tests will be used for data not conforming to normal distribution. Counting data will be analyzed using the χ^2^ test, and rank sum test for rank data. Correlation analysis between two variables will use linear correlation for bivariate normal distribution data and Spearman rank correlation for non-bivariate normal distribution or graded data. A significance level of *P* < 0.05 will be considered statistically significant. The study will also provide statistical descriptions of adverse events, adverse reactions, dropout rates, and rejections during treatment.

### 2.8 Quality control

In order to guarantee the quality of trial, all investigators are asked to attend special training includes how to fill in the Case Report Form, how to record adverse reactions, how to teach the patients to take medicine, and other details of this trial.

## 3 Discussion

Fatigue, as a concurrent symptom of UC, is frequently overlooked due to its intricate and subjective nature, posing challenges for medical staff. Nevertheless, fatigue not only exacerbates UC symptoms but also significantly impacts patients' quality of life. Clinicians should prioritize attention to the fatigue symptoms experienced by patients with UC to ensure comprehensive and effective care [23].

The etiology of fatigue in UC remains unclear. Drawing insights from research on fatigue mechanisms in other diseases, it is proposed that fatigue may result from various factors, primarily associated with the activation of the immune system, disease activity, and the release of pro-inflammatory factors [8,24,25]. Dysbiosis of gut microflora, a known contributor to gut inflammation, is now implicated in regulating fatigue through the gut–brain axis, which involves two-way communication between the gastrointestinal tract and central nervous system and gut microbes and the immune system [26]. Tryptophan metabolites [27] and polyphenol metabolites [28] have shown promise in alleviating fatigue. A prospective study suggested that microbiome- and metabolite-directed therapies reduce fatigue and depression in patients with IBD [29].

The multifaceted nature of fatigue in UC suggests the existence of different subtypes, requiring tailored intervention measures.

Despite advancements in clinical research, addressing nutrient deficiencies and using immunomodulators or biological agents to reduce systemic inflammation may not entirely alleviate UC fatigue. Studies indicate that biopharmaceuticals and small molecular drugs used for IBD, including ustekinumab, infliximab, natalizumab, tofacitinib, and upatinib, have limited efficacy in relieving fatigue [30]. Even selective JAK1 inhibitors like upatinib, while showing a significant effect on UC fatigue through long-term maintenance, lack clarity on the extent of their impact [31]. Despite the potential connection between nutrient deficiency and fatigue, there is no evidence supporting fatigue improvement in the long-term maintenance of 300 mg thiamine therapy [32], excessive use of vitamin B12 [33], and maltol iron [34] supplementation in IBD fatigue.

TCM exhibits unique advantages in combating fatigue by regulating gut microbes, protecting nerve centers, enhancing antioxidant enzyme activity, reducing metabolite accumulation, and influencing energy metabolism [35]. The JPQC prescription, designed to invigorate the spleen and replenish qi, primarily employs Qi-tonifying herbs to restore spleen Qi. Notably, Qi-tonifying herbs have demonstrated the ability to regulate intestinal flora, restoring diversity and balance [36]. Clinical studies have demonstrated that the combination of JPQC with mesalazine exerts a distinctive synergistic mechanism on the intestinal flora and metabolites of patients with ulcerative colitis (UC) by downregulating erysipelatoclostridium and decreasing the levels of 4-pyrimidine methanamine (hydrochloride), hydroxyprolyl-isoleucine, and L-glutamate. Notably, these three metabolites play a role in anti-inflammatory processes and contribute to enhanced amino acid metabolism [37]. The investigation into whether JPQC further influences UC with fatigue by regulating the disorder of intestinal flora and its metabolites presents an intriguing and crucial research topic.

The primary aim of our study is to explore the impact of JPQC on the intestinal flora and metabolites of patients with UC with spleen deficiency damp-heat type and fatigue. The study aims to investigate the clinical effects of JPQC on patients with UC with spleen deficiency and dampness-heat syndrome accompanied by fatigue, along with its regulatory mechanism on intestinal flora and metabolites. This research will contribute valuable insights into TCM's role in managing UC-related fatigue and may pave the way for novel therapeutic approaches.

## CRediT authorship contribution statement

**Zi-Xuan Liu:** Writing – review & editing, Writing – original draft. **Xiao-Yan Liu:** Writing – review & editing, Writing – original draft. **Wei-wei Tan:** Writing – review & editing. **Wei-Bing Zhang:** Writing – review & editing. **Ya-Li Zhang:** Writing – original draft, Methodology. **Lie Zheng:** Writing – original draft, Formal analysis. **Yan-Cheng Dai:** Writing – review & editing, Writing – original draft, Methodology, Formal analysis.

## Ethics approval and consent to participate

We confirm that this trial will be conducted in accordance with the principles of the Declaration of Helsinki.

This study was approved by the Ethics Committee of Shanghai Hospital of Integrated Traditional Chinese and Western Medicine affiliated with Shanghai University of Traditional Chinese Medicine (2022-053-1). All participate will be required to sign written informed consent.

## Funding

This study was funded by the grants from the 10.13039/501100001809National Natural Science Foundation of China, No. 81873253; the 10.13039/100007219Shanghai Natural Science Foundation, No. 22ZR1458800; the scientific research Project Plan of 10.13039/100017950Shanghai Municipal Health Commission, No. 202240385; and the Xinglin Scholar Program of 10.13039/501100010876Shanghai University of Traditional Chinese Medicine, No. [2020]23; Hongkou District 10.13039/100018696Health Committee, No. HKZK2020A01.

## Declaration of competing interest

The authors declare that they have no known competing financial interests or personal relationships that could have appeared to influence the work reported in this paper.

## Data Availability

No data was used for the research described in the article.
